# Supplementary material for: Engineering tRNA abundances for synthetic cellular systems
Source: Nat Commun. 2023 Jul 31;14:4594. doi: 10.1038/s41467-023-40199-9 (PMC10390467; doi:10.1038/s41467-023-40199-9)
Supplement: Supplementary file 1 — Supplementary Information [file 41467_2023_40199_MOESM1_ESM.pdf]

Supplement to: Engineering tRNA abundances for synthetic cellular systems

Akshay J. Maheshwari[1,3], Jonathan Calles[1,3], Sean K. Waterton[2], Drew Endy[1,*]

[1] Department of Bioengineering, Stanford University, Stanford, CA 94305, USA
[2] Department of Biology, Stanford University, Stanford, CA 94305, USA
[3] equal contributors
[*]Correspondence: endy@stanford.edu (D.E.)

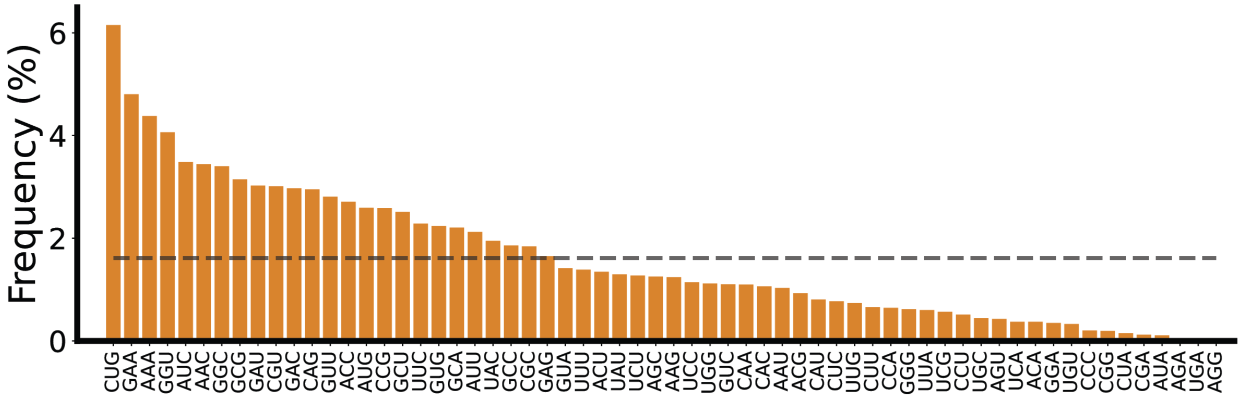

Figure S1. Codons sorted by relative usage in the *E. coli* transcriptome. Dashed line corresponds to uniform usage across all codons. Source data are provided via a Zenodo repository (10.5281/zenodo.7953836).

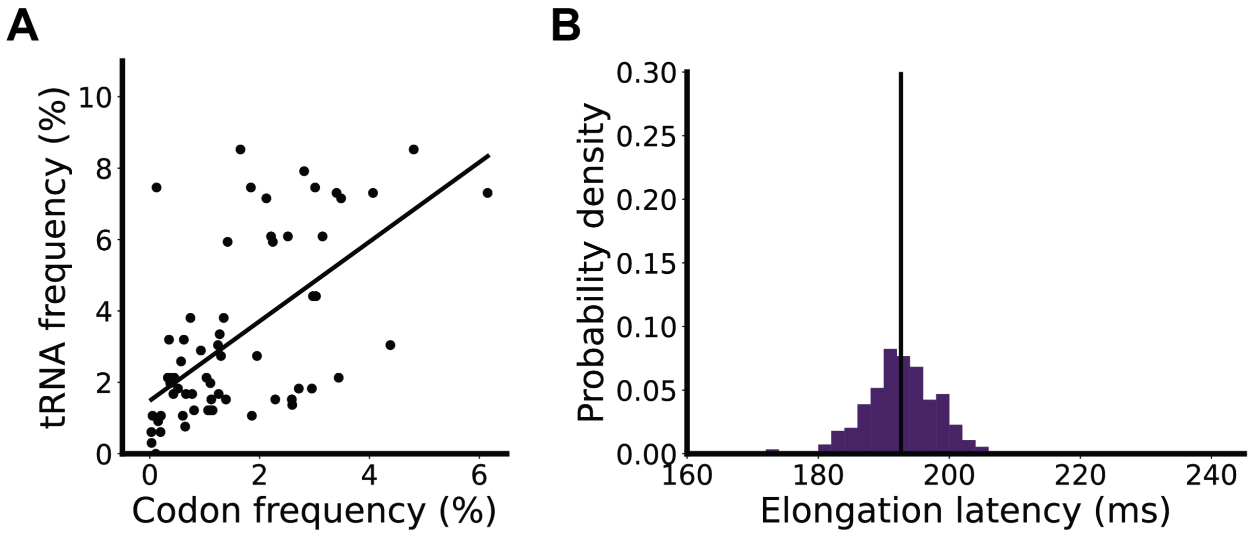

Figure S2. (a) Fraction of cognate tRNA per codon (each codon is represented by its relative usage in the transcriptome) for wild-type *E. coli*. Slope of fit = 1.12. **(b)** The per-transcript elongation latency distribution (average, black line) for wild-type *E. coli*. Source data are provided via a Zenodo repository (10.5281/zenodo.7953836).

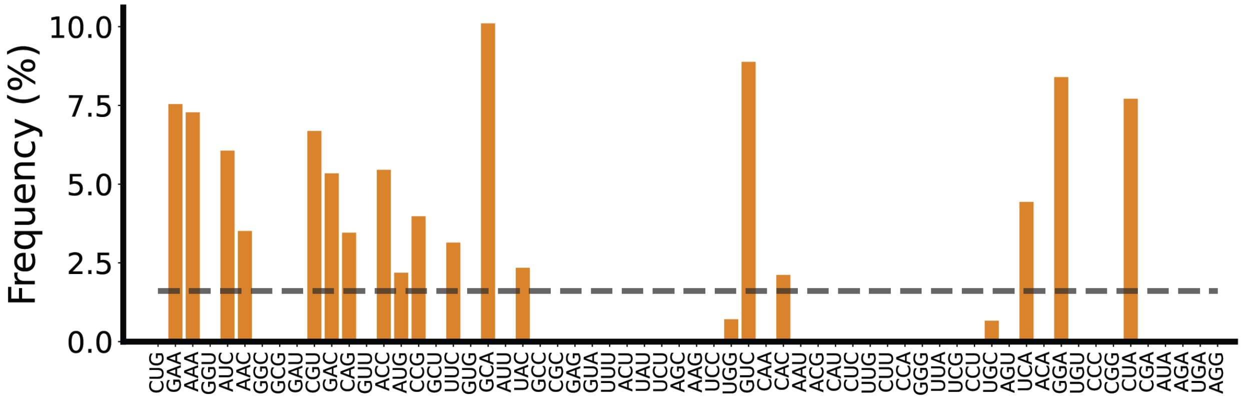

Figure S3. Codons sorted by relative usage in the computationally designed RED-20 reduced-genome transcriptome. Dashed line corresponds to uniform usage across all utilized codons. Source data are provided via a Zenodo repository (10.5281/zenodo.7953836).

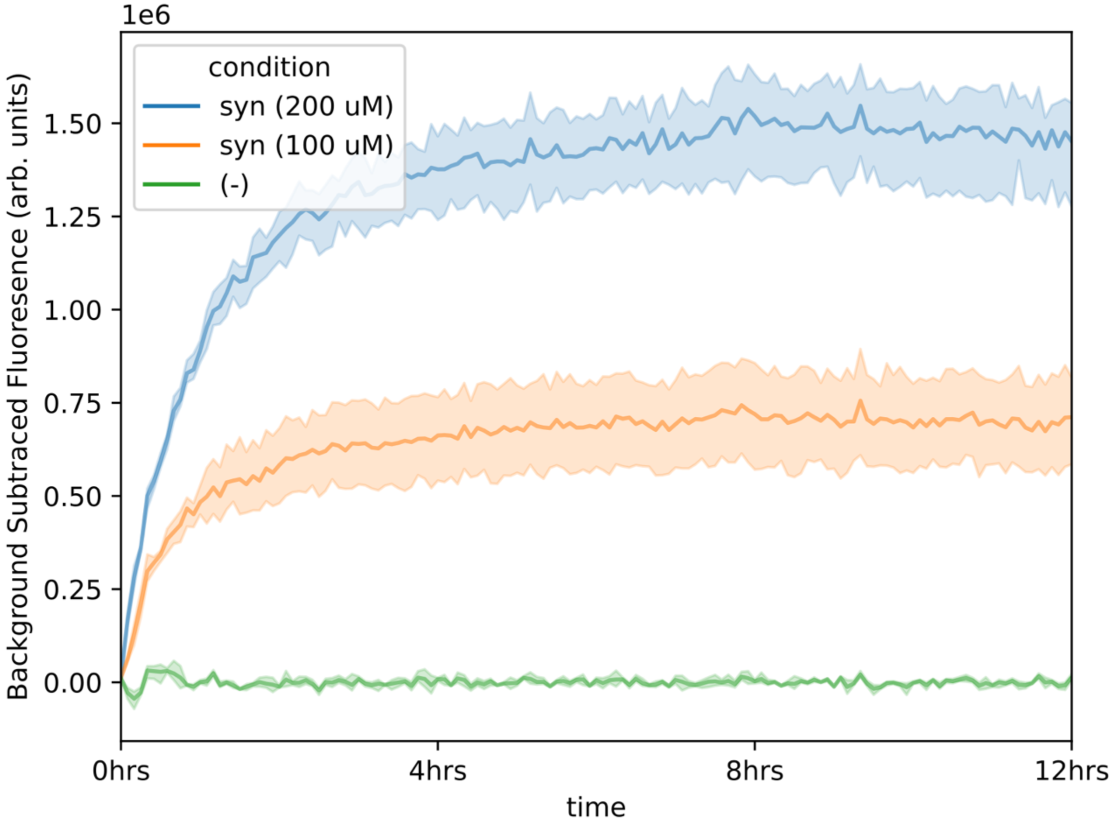

Figure S4: Measuring protein expression from lower concentrations of synthetic tRNAs. Mean fluorescence vs time traces (solid lines) for *in vitro* protein expression of a RED20-encoded GFP reporter using synthetic tRNAs. We varied total tRNA concentration across conditions (200 µM total tRNAs, blue; 100 µM total tRNAs, orange; 0 µM total tRNAs, green). Shaded regions represent the 95% confidence interval in the estimate of the mean for each condition (n=3 technical replicates). We demonstrated expression reliably above the noise floor for our experimental setup for both 200 µM and 100 µM total tRNAs, suggesting that we could reduce tRNA concentration by at least 10x relative to the previously reported concentration (1050 µM total tRNA; see Calles *et al.* 2019). Source data are provided via a Zenodo repository (10.5281/zenodo.7953836).

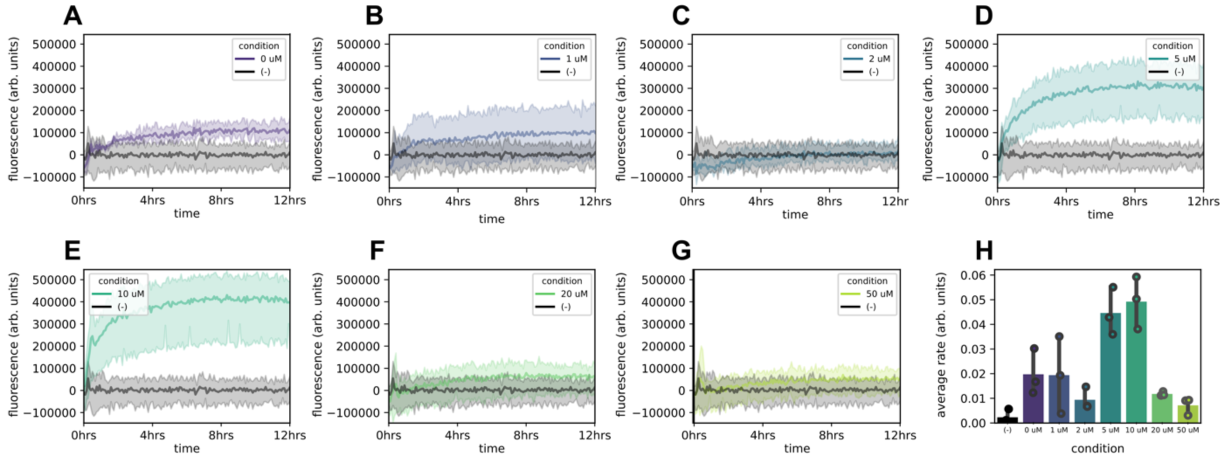

Figure S5: Calibrating init-Met concentration in TINA. Fluorescence vs time traces for *in vitro* protein expression of a RED20-encoded GFP reporter using 200 µM elongator tRNAs in a uniform distribution and varying amounts of initiator tRNA, ranging between **(a)** 0 uM (purple), **(b)** 1 µM (indigo), **(c)** 2 µM (slate), **(d)** 5µM (blue), **(e)**10 µM (blue green), **(f)** 20 µM (green), **(g)** and 50 µM (lime green). Black represents PUREΔtRNA (labelled "(-)") and is used to calculate background fluorescence throughout. Shaded regions represent the 95% confidence interval in the estimate of the mean for each condition (n=3 technical replicates). **(h)** Calculated protein production rates for each initiator tRNA concentration (n=3 technical replicates). Error bars represent the 95% confidence interval in the estimate of the mean within condition. Colors as (a) through (g). Color names subjective. Source data are provided via a Zenodo repository (10.5281/zenodo.7953836).

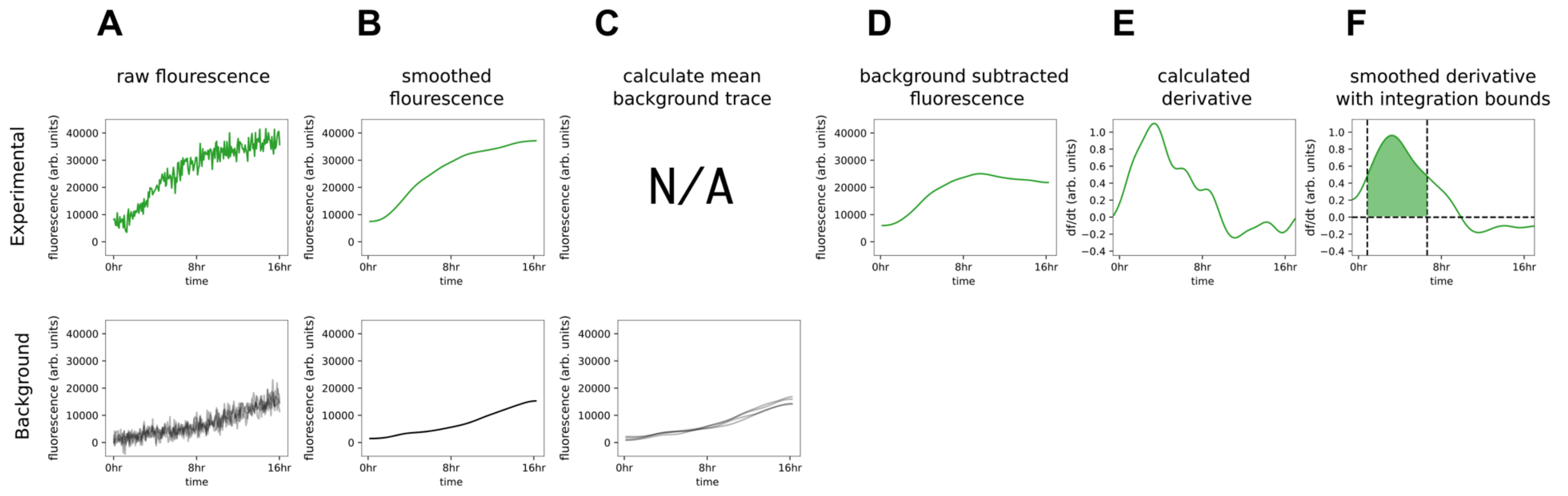

Figure S6: Calculating protein production rates from fluorescence traces. (a) Raw fluorescence traces as taken from the plate reader. Above (green) represents a single trace with 200 μM elongator tRNAs in a uniform distribution. Below (black) represents five traces from PUREΔtRNA, used in this experiment as a negative control and measurement of background fluorescence. **(b)** Traces as in (a) after smoothing with a Gaussian kernel (σ = 50 min). **(c)** Calculated average background trace (below, black). This trace is the result of averaging the five PUREΔtRNA traces in (b) at each timepoint **(d)** Smoothed fluorescence trace from (b) with mean background signal subtracted at each timepoint. **(e)** Derivative of trace from (d) numerically calculated using numpy.gradient method. **(f)** Smoothed derivative from (e). Shaded region represents values we average over to calculate the protein production rate. Dashed lines represent the bounds of the region over which we average the derivative. To find these bounds for any given trace, we first found the maximum value of the derivative and the timepoint at which that maximum occurs. We then defined a threshold derivative value for each trace as some fraction of the max derivative value (here, 50% max derivative). We finally defined the bounds as the timepoints immediately before and after the max derivative timepoint when the derivative rises above and drops below that threshold, respectively. Source data are provided via a Zenodo repository (10.5281/zenodo.7953836).

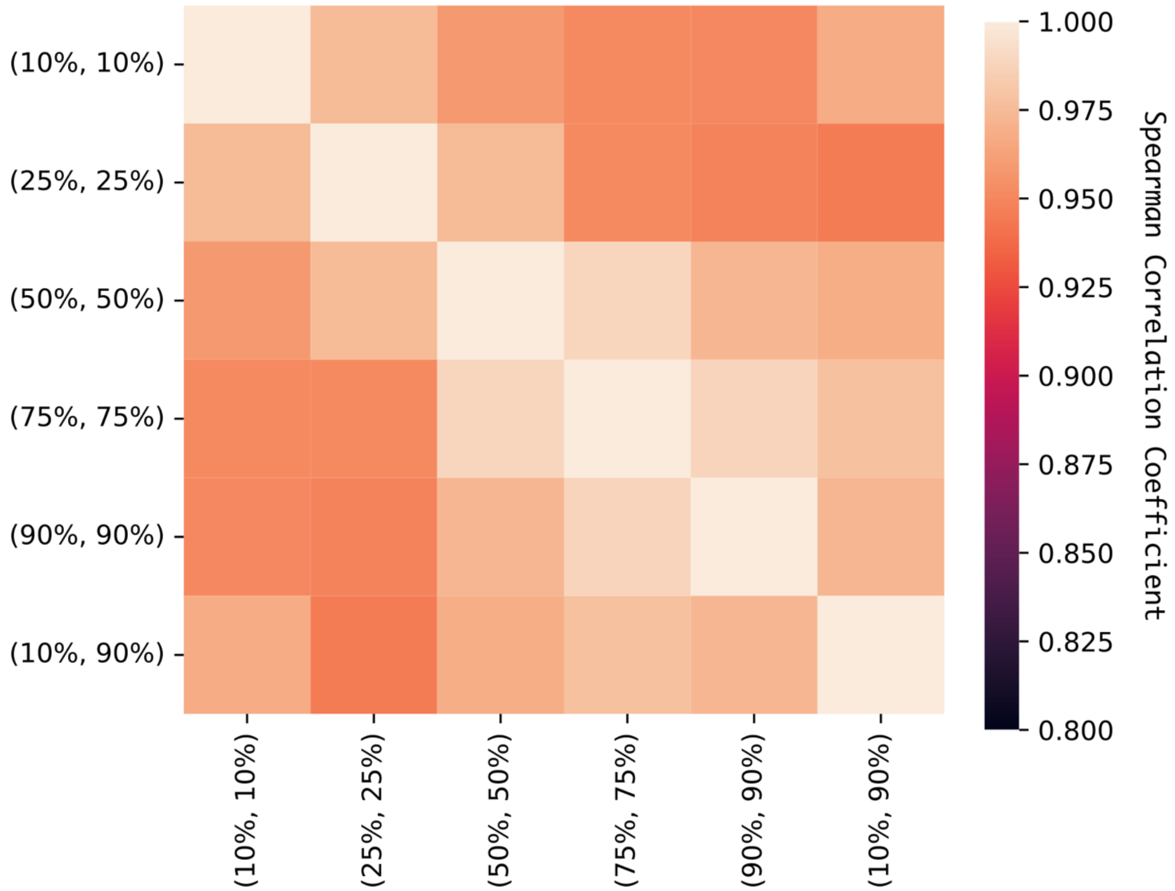

Figure S7: Normalized protein production rates strongly correlated across choice of bounds. Spearman correlation coefficients for normalized protein production rates across a wide range of integration bounds. Color bar represents pairwise Spearman correlation coefficients across conditions. For each set of integration bounds, we recalculated the protein production rate for each reaction used in Figure 5 using those bounds. We then calculated the correlation coefficient between every pair of those sets of integration bounds. Source data are provided via a Zenodo repository (10.5281/zenodo.7953836).

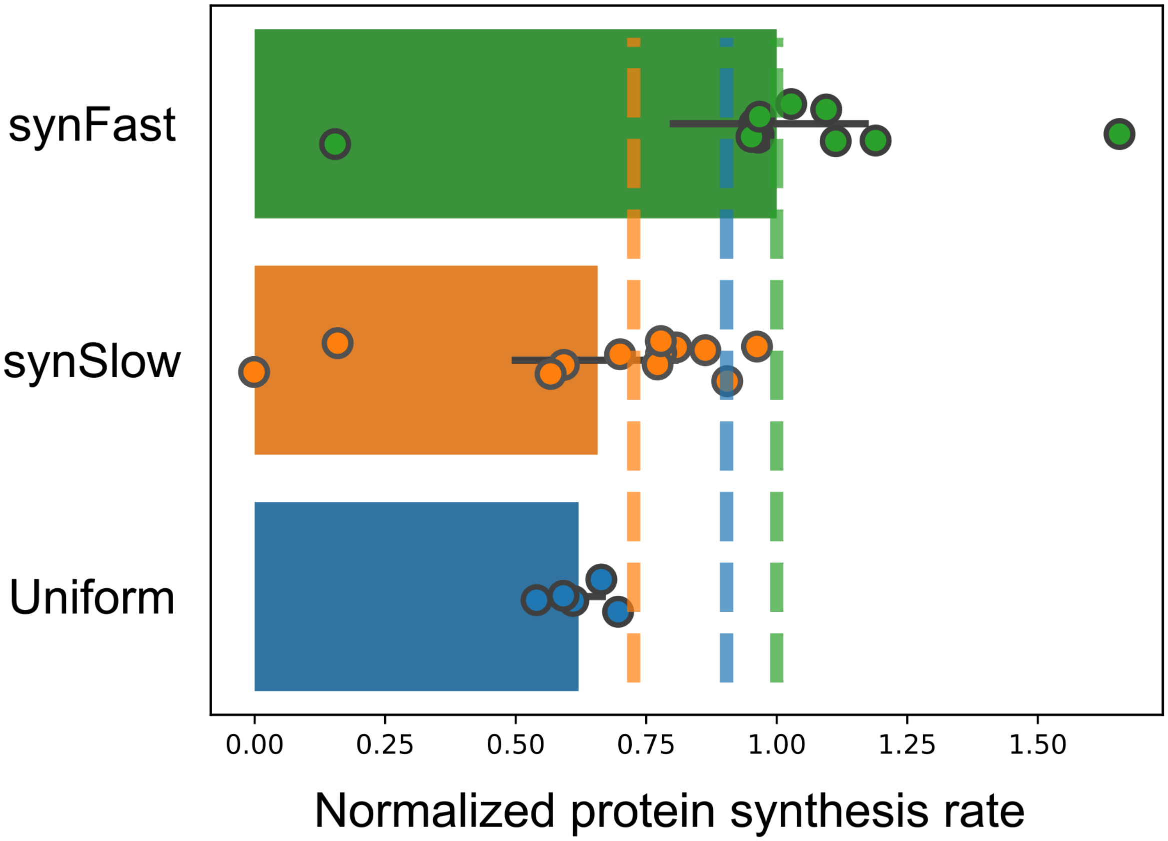

Figure S8: Normalized protein synthesis rate vs condition pooled across tRNA batches. Experimentally derived protein synthesis rates for three tRNA distributions: synFast (green, n=12), synSlow (orange, n=12), and uniform distribution (blue, n=5). Each data point is normalized within tRNA batch to the average synFast protein production rate for that given tRNA batch. Individual points represent normalized production values from a single fluorescence trace. Error bars represent 95% confidence interval in the estimate of the mean. Predicted protein synthesis rates for each tRNA distribution as shown (vertical dashed lines, color matched to bars). Source data are provided via a Zenodo repository (10.5281/zenodo.7953836).

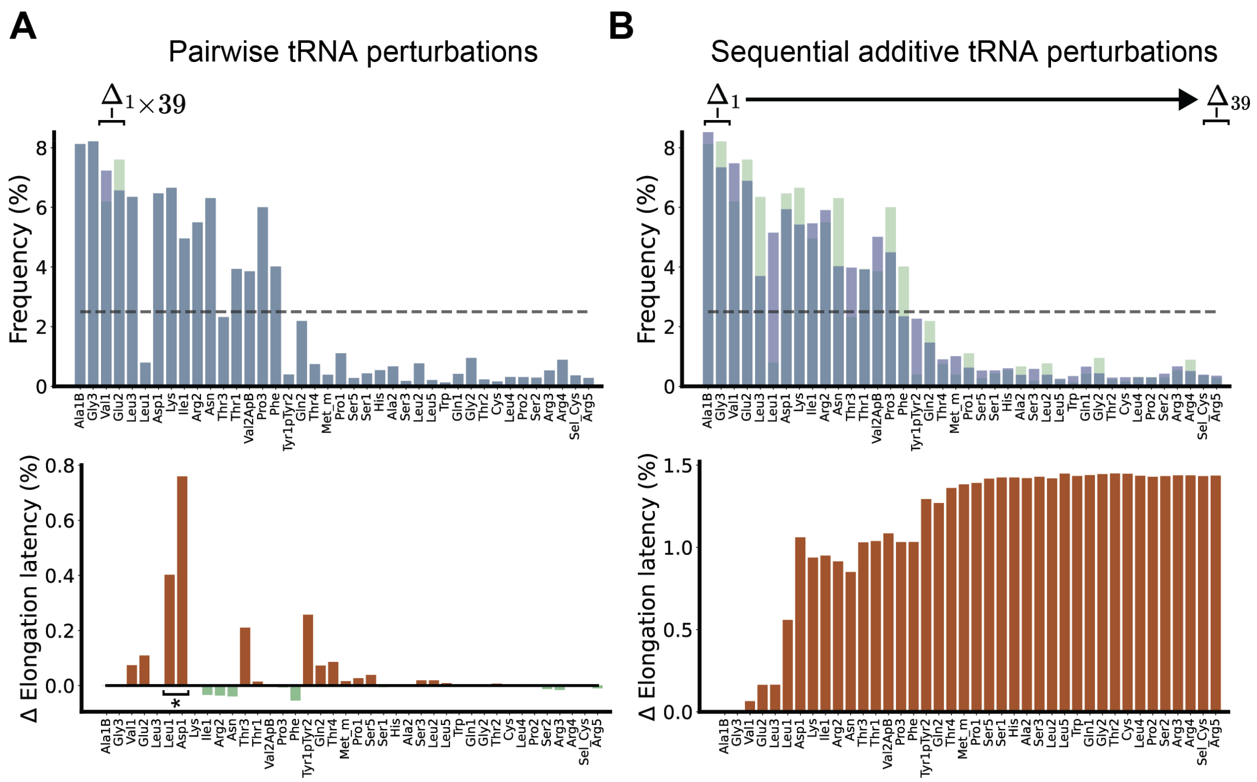

Figure S9. Most monotonic perturbations away from the CD-CAD-discovered non-monotonic fast tRNA distribution lead to slower elongation latencies. A. Pairwise tRNA perturbations across nearest neighbor tRNA ordered by corresponding codon frequency. In each of the 39 pairs the more weighted tRNA is set to be 10% more abundant than the less weighted tRNA, i.e., towards a more monotonic distribution. Top: An example of a single pairwise tRNA perturbation in which relative abundance is moved from Glu2 to Val1 such that the relative abundance of Val1 is 10% higher than Glu2 and no other tRNA are changed. Both the original and perturbed distributions are shown overlaid (green and blue, respectively). Bottom: Predicted change in average elongation latency relative to baseline for each pairwise tRNA (brown and green color of bars represent whether the change in elongation latency is positive or negative, respectively; average computed from N=30 runs). Perturbation of the relative abundance of Leu1 results in the largest change in elongation latency (marked with an asterisk). **B.** Sequential addition of each of the 39 pairwise tRNA perturbations. Top: The final 39-pairwise tRNA perturbed distribution (blue) overlaid on the original CD-CAD discovered tRNA distribution (green). Bottom: Predicted change in average elongation latency relative to baseline for each additional pairwise perturbation (e.g., bar at Gly3 corresponds to the elongation latency of the CD-CAD-found distribution with one pairwise perturbation and the bar at Arg5 is the elongation latency of the CD-CAD-found distribution with 39 sequential pairwise perturbations; average computed from N=3000 runs). Source data are provided via a Zenodo repository (10.5281/zenodo.7953836).

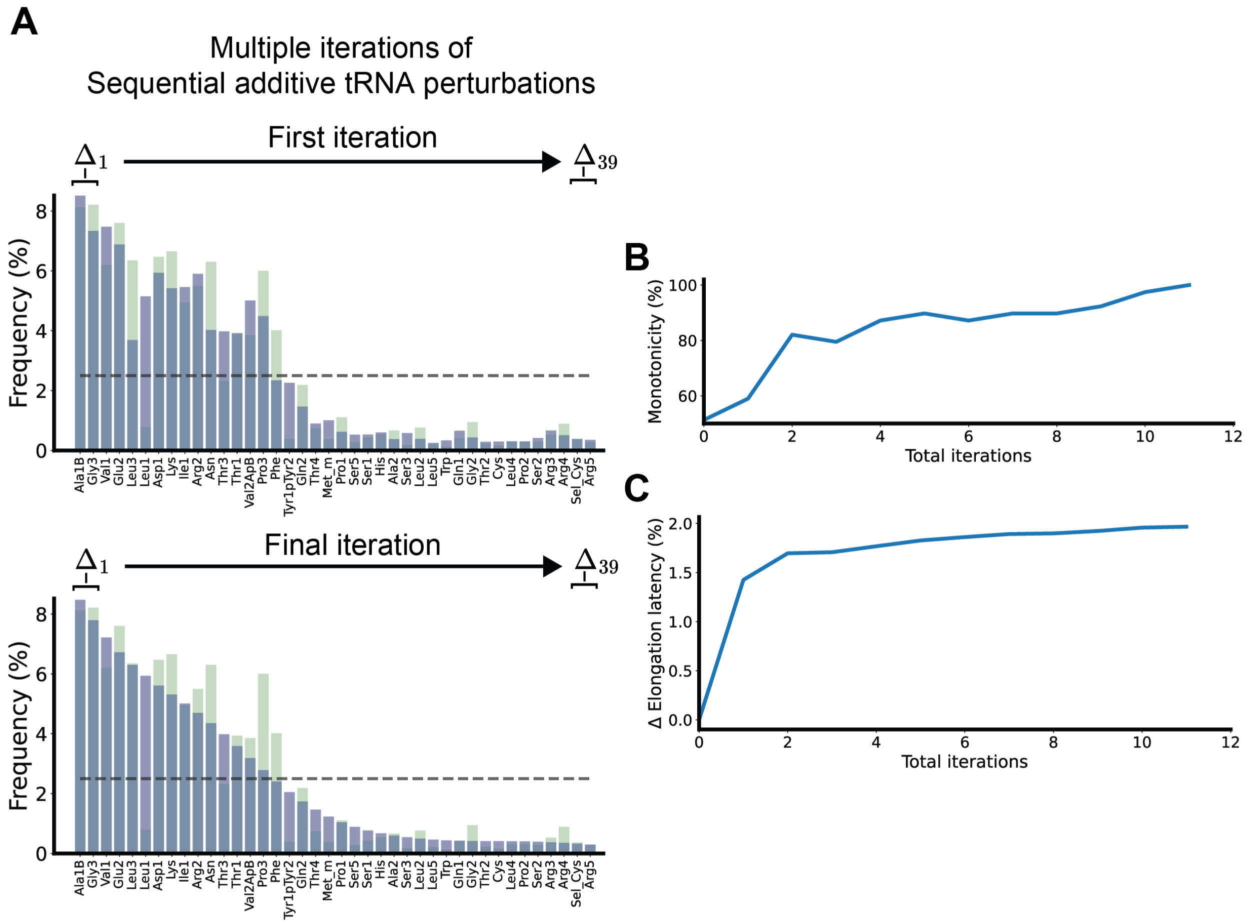

Figure S10. Iteratively increasing the monotonicity of the CD-CAD-discovered non-monotonic fast tRNA distribution leads to slower elongation latencies. A. The first and final iterations of 39-sequential pairwise tRNA perturbations, with tRNA ordered by relative abundance of corresponding codons (perturbed distribution in blue, baseline CD-CAD discovered distribution in green). **B.** Each additional 39-sequential pairwise tRNA perturbation iteration leads to higher monotonicity of tRNA distributions, quantified as the percent of pairwise tRNA neighbors in which the tRNA corresponding to higher codon frequency has greater relative abundance. **C.** Average elongation latency is slower compared to baseline elongation latency at each iteration and overall becomes slower with increasing monotonicity (each elongation latency iteration data point computed from N=3000 runs). Source data are provided via a Zenodo repository (10.5281/zenodo.7953836).

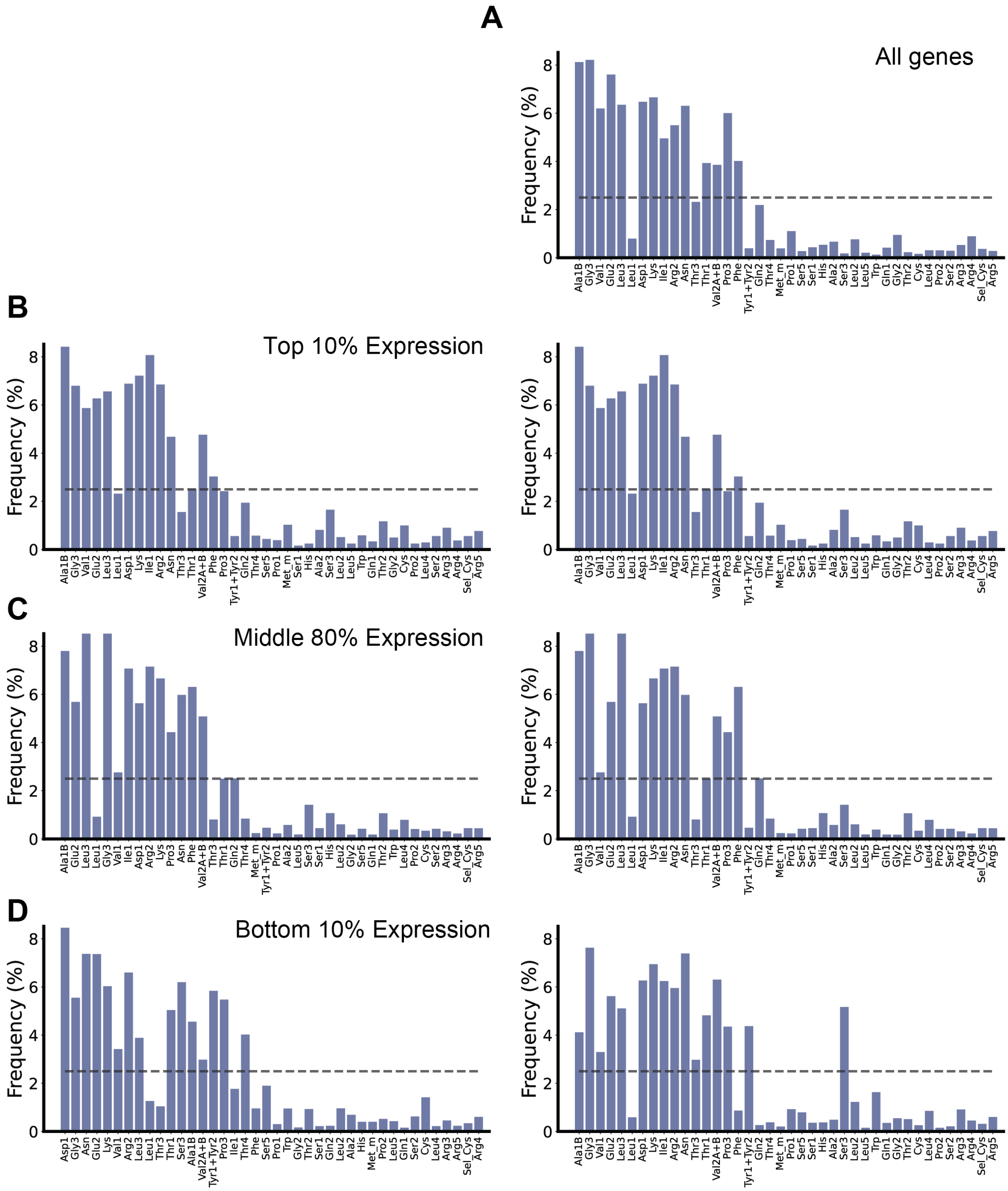

Figure S11. Optimizing tRNA distributions for fast translation of gene subpopulations stratified by expression level reveals distinct component distributions underlying the non-monotonic overall tRNA relative abundance distribution. A. Baseline CD-CAD-optimized relative abundance of tRNA for fast expression across all genes. **B, C, D.** CD-CAD optimization for fast tRNA distributions across high expression, intermediate expression, and low expression gene subsets. Left: tRNA distributions rank-ordered by codon-usage within gene subsets. Right: tRNA distributions ordered in the same way as the baseline tRNA distribution. Source data are provided via a Zenodo repository (10.5281/zenodo.7953836).

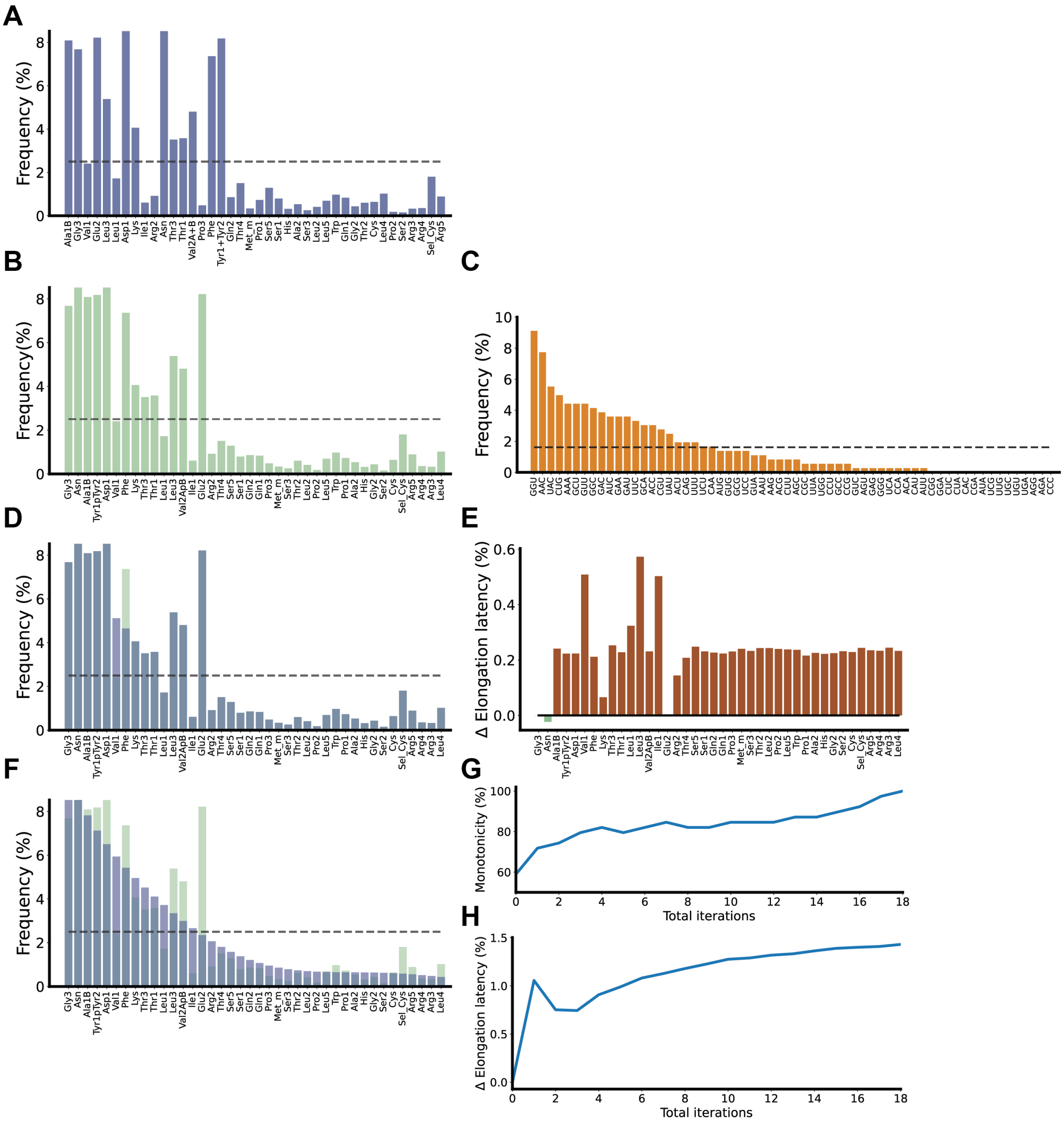

Figure S12. CAD-optimization and sensitivity analysis of the single high expressing gene ompF reveals non-monotonicity intrinsic to degeneracy of translation. A. Relative tRNA abundance produced by CD-CAD optimized for fast expression of ompF, with tRNA ordered by overall transcriptome codon abundance. **B.** The same relative tRNA abundance reordered by codon usage in ompF. **C.** Codon usage in ompF. **D.** An example of pairwise perturbation of tRNA relative abundance (perturbed distribution in blue, baseline CD-CAD discovered distribution in green). **E.** Changes in average elongation latency resulting from pairwise perturbation across every neighboring pair of tRNA ordered by codon usage in ompF (average computed from N=3000 runs of each perturbation). **F.** Fully monotonic tRNA distribution (blue) resulting from iteratively making the original CD-CAD discovered tRNA distribution (green) more monotonic. **G.** Monotonicity increasing over iterations (quantified as the percent

of pairwise tRNA neighbors in which the tRNA corresponding to higher codon frequency has greater relative abundance). **H.** Predicted average elongation latency is slower compared to baseline with increasing monotonicity (each elongation latency iteration data point computed from N=3000 runs). Source data are provided via a Zenodo repository (10.5281/zenodo.7953836).

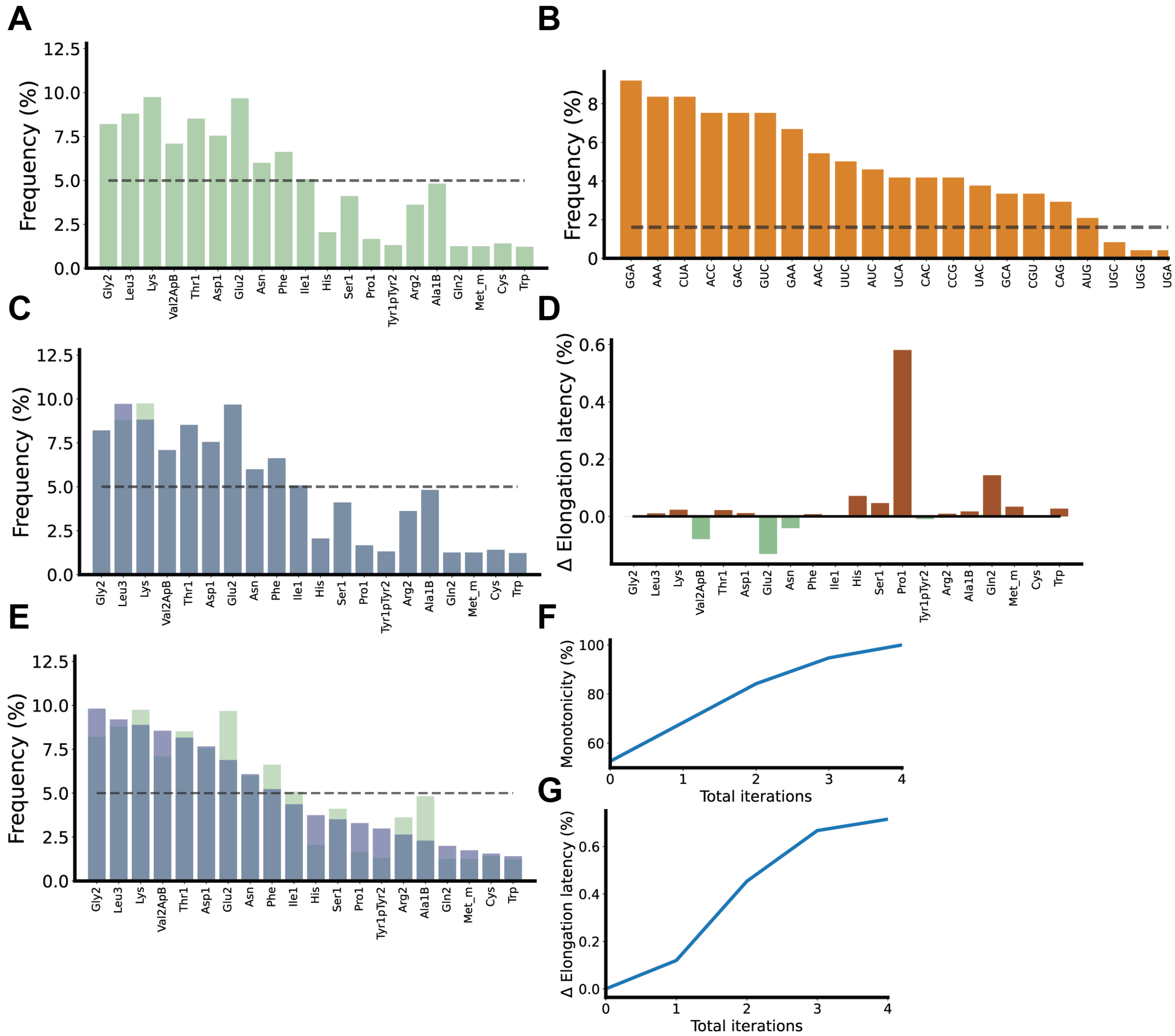

Figure S13. CAD optimization and sensitivity analysis of RED20-encoded GFP in a minimal tRNA system reveals how expression in a non-degenerate translation system can behave optimally even with a monotonic tRNA abundance distribution. A. Relative tRNA abundance produced by CD-CAD optimized for fast expression of RED-20 GFP in a minimal tRNA system. **B.** Codon usage in RED20-encoded GFP. **C.** An example of pairwise perturbation of tRNA abundance (perturbed distribution in blue, baseline CD-CAD discovered distribution in green). **D.** Changes in elongation latency resulting from pairwise perturbation across every neighboring pair of tRNA ordered by codon usage in RED20-encoded GFP (average computed from N=3000 runs of each perturbation). **E.** Fully monotonic tRNA distribution (blue) resulting from iteratively making the original CD-CAD discovered tRNA distribution (green) more monotonic. **F.** Monotonicity increasing over iterations (quantified as the percent of pairwise tRNA neighbors in which the tRNA corresponding to higher codon-frequency has greater relative abundance). **G.** Predicted average elongation latency is only marginally slower overall compared to baseline with increasing monotonicity (each elongation latency iteration data point computed from N=3000 runs). Source data are provided via a Zenodo repository (10.5281/zenodo.7953836).

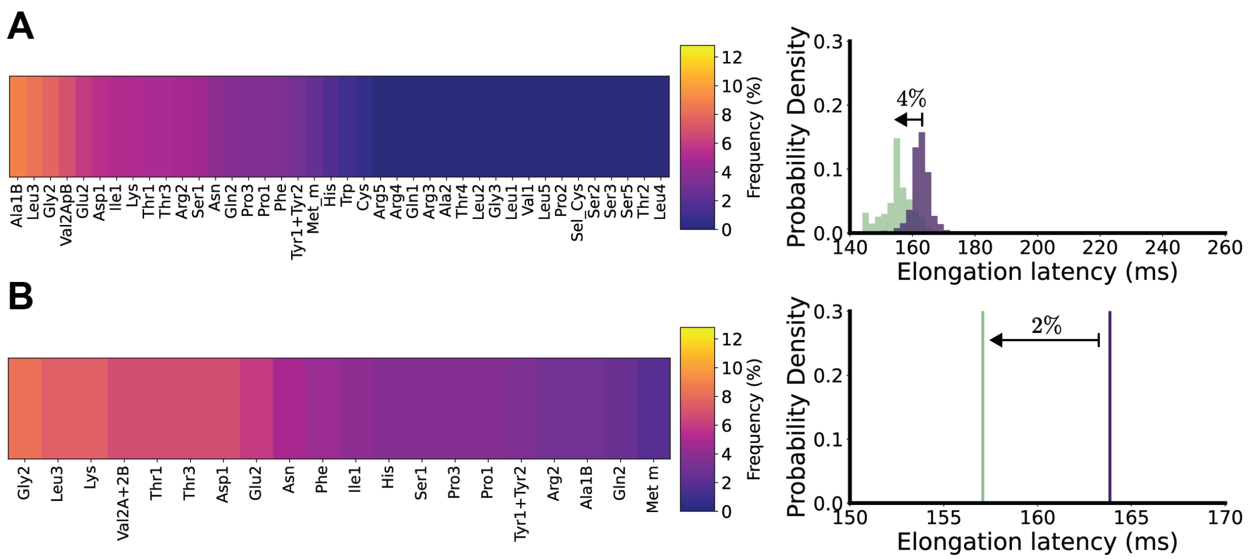

Figure S14. CAD-produced fast tRNA distributions perform better than codon-weighted distributions for both the RED20 codon-reduced transcriptome and RED20-encoded GFP. A. Left: Codon-weighted distribution of the reduced set of tRNA for expression of the RED20 codon-reduced transcriptome (color bar, percentages). Right: Per-transcript elongation latency distribution for the CAD-produced fast tRNA distribution (green) vs. codon-weighted (purple). **B.** Left: Codon-weighted distribution of the reduced set of tRNA for expression of RED20-encoded GFP. Right: Elongation latency for the CAD-produced fast tRNA distribution (green) vs. codon-weighted (purple). Source data are provided via a Zenodo repository (10.5281/zenodo.7953836).

**Population size**	100
**# candidates recombining per generation**	10
**Recombination strategy**	Random pairs, random indices
**# candidates removed per generation**	10
**Recombination rate**	0.2
**Mutation rate**	0.1

Table S1. Genetic algorithm simulation parameters.

Amino Acid	Anticodon	RNA Sequence
Phenylalanine	GAA	GCCCGGAUAGCUCAGUCGGUAGAGCAGGGGAUUGAAAAUCCCCGUGUCCUUGGUUCGAUUCCGAGUCCGGGCACCA
Leucine	UAG	GCGGGAGUGGCGAAAUUGGUAGACGCACCAGAUUUAGGUUCUGGCGCCGCAAGGUGUGCGAGUUCAAGUCUCGCCUCCCGCACCA
fMethionine	CAU	CGCGGGGUGGAGCAGCCUGGUAGCUCGUCGGGCUCAUAACCCGAAGGUCGUCGGUUCAAAUCCGGCCCCCGCAACCA
Methionine	CAU	GGCUACGUAGCUCAGUUGGUUAGAGCACAUCACUCAUAAUGAUGGGGUCACAGGUUCGAAUCCCGUCGUAGCCACCA
Valine	GAC	GCGUUCAUAGCUCAGUUGGUUAGAGCACCACCUUGACAUGGUGGGGGUCGUUGGUUCGAGUCCAAUUGAACGCACCA
Serine	UGA	GGAAGUGUGGCCGAGCGGUUGAAGGCACCGGUCUUGAAAACCGGCGACCCGAAAGGGUUCCAGAGUUCGAAUCUCUGCGCUUCCGCCA
Proline	CGG	CGGUGAUUGGCGCAGCCUGGUAGCGCACUUCGUUCGGGACGAAGGGGUCGGAGGUUCGAAUCCUCUAUCACCGACCA
Threonine	GGU	GCUGAUAUAGCUCAGUUGGUAGAGCGCACCCUUGGUAAGGGUGAGGUCGGCAGUUCGAAUCUGCCUAUCAGCACCA
Alanine	UGC	GGGGCUAUAGCUCAGCUGGGAGAGCGCCUGCUUUGCACGCAGGAGGUCUGCGGUUCGAUCCCGCAUAGCUCCACCA
Tyrosine	GUA	GGUGGGGUUCCCGAGCGGCCAAAGGGAGCAGACUGUAAAUCUGCCGUCACAGACUUCGAAGGUUCGAAUCCUUCCCCCACCACCA
Glutamine	CUG	UGGGGUAUCGCCAAGCGGUAAGGCACCGGAUUCUGAUUCCGGCAUUCCGAGGUUCGAAUCCUCGUACCCCAGCCA
Lysine	UUU	GGGUCGUUAGCUCAGUUGGUAGAGCAGUUGACUUUUAAUCAAUUGGUCGCAGGUUCGAAUCCUGCACGACCCACCA
Aspartate	GUC	GGAGCGGUAGUUCAGUCGGUUAGAAUACCUGCCUGUCACGCAGGGGGUCGCGGGUUCGAGUCCCGUCCGUUCCGCCA
Tryptophan	CCA	AGGGGCGUAGUUCAAUUGGUAGAGCACCGGUCUCCAAAACCGGGUGUUGGGAGUUCGAGUCUCUCCGCCCCUGCCA
Arginine	ACG	GCAUCCGUAGCUCAGCUGGAUAGAGUACUCGGCUACGAACCGAGCGGUCGGAGGUUCGAAUCCUCCCGGAUGCACCA
Glycine	UCC	GCGGGCAUCGUAUAAUGGCUAUUACCUCAGCCUUCCAAGCUGAUGAUGCGGGUUCGAUUCCCGCUGCCCGCUCCA
Isoleucine	GAU	AGGCUUGUAGCUCAGGUGGUUAGAGCGCACCCCUGAUAAGGGUGAGGUCGGUGGUUCAAGUCCACUCAGGCCUACCA
Histidine	GUG	GGUGGCUAUAGCUCAGUUGGUAGAGCCCUGGAUUGUGAUUCCAGUUGUCGUGGGUUCGAAUCCCAUUAGCCACCCCA
Asparagine	GUU	UCCUCUGUAGUUCAGUCGGUAGAACGGCGGACUGUUAAUCCGUAUGUCACUGGUUCGAGUCCAGUCAGAGGAGCCA
Glutamate	UUC	GUCCCCUUCGUCUAGAGGCCCAGGACACCGCCCUUUCACGGCGGUAACAGGGGUUCGAAUCCCCUAGGGGACGCCA
Cysteine	GCA	GGCGCGUUAACAAAGCGGUUAUGUAGCGGAUUGCAAAUCCGUCUAGUCCGGUUCGACUCCGGAACGCGCCUCCA

Table S2: tRNA sequences used in TINA *in vitro* expression system. RNA sequences for each of the 21 tRNAs used in this work. “fMethionine” represents the initiator tRNA, where “Methionine” represents the elongator tRNA associated with Methionine.

	uniform	synFast	synSlow
Ala	10.00	4.56	25.65
Arg	10.00	3.14	4.03
Asn	10.00	13.78	4.04
Asp	10.00	15.29	2.92
Cys	10.00	2.44	24.26
Gln	10.00	9.44	25.12
Glu	10.00	14.63	2.95
Gly	10.00	20.39	3.11
His	10.00	7.86	3.01
Ile	10.00	8.30	3.87
Leu	10.00	14.98	3.07
Lys	10.00	20.51	3.11
Met	10.00	2.44	23.90
Phe	10.00	9.07	4.10
Pro	10.00	2.44	25.65
Ser	10.00	12.73	3.99
Thr	10.00	16.48	3.13
Trp	10.00	2.44	25.50
Tyr	10.00	6.95	4.41
Val	10.00	12.13	4.17
init-tRNA	10.00	10.00	10.00

Table S3: Physical tRNA concentrations used for *in vitro* protein expression experiments. All concentrations are in units of μM.
