# Supplementary material for: Engineering tRNA abundances for synthetic cellular systems
Source: Nat Commun. 2023 Jul 31;14:4594. doi: 10.1038/s41467-023-40199-9 (PMC10390467; doi:10.1038/s41467-023-40199-9)
Supplement: Supplementary file 3 — Reporting Summary [file 41467_2023_40199_MOESM3_ESM.pdf]

nature portfolio

Corresponding author(s): Drew Endy

Last updated by author(s): Jun 29, 2023

Reporting Summary

Nature Portfolio wishes to improve the reproducibility of the work that we publish. This form provides structure for consistency and transparency in reporting. For further information on Nature Portfolio policies, see our Editorial Policies and the Editorial Policy Checklist.

Statistics

For all statistical analyses, confirm that the following items are present in the figure legend, table legend, main text, or Methods section.

n/a	Confirmed	
☐	☒	The exact sample size (n) for each experimental group/condition, given as a discrete number and unit of measurement
☐	☒	A statement on whether measurements were taken from distinct samples or whether the same sample was measured repeatedly
☐	☒	The statistical test(s) used AND whether they are one- or two-sided<br>*Only common tests should be described solely by name; describe more complex techniques in the Methods section.*
☐	☒	A description of all covariates tested
☐	☒	A description of any assumptions or corrections, such as tests of normality and adjustment for multiple comparisons
☐	☒	A full description of the statistical parameters including central tendency (e.g. means) or other basic estimates (e.g. regression coefficient) AND variation (e.g. standard deviation) or associated estimates of uncertainty (e.g. confidence intervals)
☐	☒	For null hypothesis testing, the test statistic (e.g. F, t, r) with confidence intervals, effect sizes, degrees of freedom and P value noted<br>*Give P values as exact values whenever suitable.*
☐	☒	For Bayesian analysis, information on the choice of priors and Markov chain Monte Carlo settings
☐	☒	For hierarchical and complex designs, identification of the appropriate level for tests and full reporting of outcomes
☐	☒	Estimates of effect sizes (e.g. Cohen's d, Pearson's r), indicating how they were calculated

Our web collection on statistics for biologists contains articles on many of the points above.

Software and code

Policy information about availability of computer code

Data collection	All computer code is available on Github (https://github.com/EndyLab/tRNACAD) and has also been deposited on Zenodo: 10.5281/zenodo.8088058 (https://zenodo.org/record/8088058). For modeling data collection, we built upon software from Maheshwari et al., 2022 (mBio) and used Python 3.7.4.
Data analysis	All computer code is available on Github (https://github.com/EndyLab/tRNACAD) and has also been deposited on Zenodo: 10.5281/zenodo.8088058 (https://zenodo.org/record/8088058). For experimental analysis, we used Python 3.10 and its scientific analysis and visualization ecosystem (numpy 1.23.5, scipy 1.9.3, pandas 1.5.2, matplotlib 3.6.2, seaborn 0.12.1). For modeling data analysis, we built upon software from Maheshwari et al., 2022 (mBio) and used Python 3.7.4.

For manuscripts utilizing custom algorithms or software that are central to the research but not yet described in published literature, software must be made available to editors and reviewers. We strongly encourage code deposition in a community repository (e.g. GitHub). See the Nature Portfolio guidelines for submitting code & software for further information.

Data

Policy information about availability of data

All manuscripts must include a data availability statement. This statement should provide the following information, where applicable:

- Accession codes, unique identifiers, or web links for publicly available datasets
- A description of any restrictions on data availability
- For clinical datasets or third party data, please ensure that the statement adheres to our policy

All input and output simulation files and all raw and processed experimental data are available via a Zenodo data repository: 10.5281/zenodo.7953836 (https://zenodo.org/record/7953836). Genome and sequence data from Ecocyc and Caglar et al., 2017 are also provided in the Zenodo repository.

Research involving human participants, their data, or biological material

Policy information about studies with human participants or human data. See also policy information about sex, gender (identity/presentation), and sexual orientation and race, ethnicity and racism.

Reporting on sex and gender	N/A
Reporting on race, ethnicity, or other socially relevant groupings	N/A
Population characteristics	N/A
Recruitment	N/A
Ethics oversight	N/A

Note that full information on the approval of the study protocol must also be provided in the manuscript.

Field-specific reporting

Please select the one below that is the best fit for your research. If you are not sure, read the appropriate sections before making your selection.

☒ Life sciences ☐ Behavioural & social sciences ☐ Ecological, evolutionary & environmental sciences

For a reference copy of the document with all sections, see nature.com/documents/nr-reporting-summary-flat.pdf

Life sciences study design

All studies must disclose on these points even when the disclosure is negative.

Sample size	Sample sizes for simulations were determined based on replicates required to distinguish between conditions. For experiments, we conducted as many replicates as possible given how much of our limiting reagent we could source (synthetic tRNAs). We did not calculate statistical power of our sample size a priori.
Data exclusions	We report all data gathered. No data were excluded.
Replication	We repeated our experiments over three technical replicates over three days. Results qualitatively agreed across days and all replicates were reported.
Randomization	All samples in this study were included in all experimental conditions (i.e., tRNA-Leu was added to synFast, synSlow, and Uniform; mutatis mutandis for all other tRNA species). Accordingly, experiments were not randomized.
Blinding	Blinding during actual experiments was not possible as the manual combination of different reagents for different experimental groups makes apparent which experimental group a particular experiment belongs to. All analysis of experimental data from different experimental groups was performed computationally and automatically without need for blinding.

Reporting for specific materials, systems and methods

We require information from authors about some types of materials, experimental systems and methods used in many studies. Here, indicate whether each material, system or method listed is relevant to your study. If you are not sure if a list item applies to your research, read the appropriate section before selecting a response.

Materials & experimental systems

n/a	Involved in the study
☒	☐ Antibodies
☒	☐ Eukaryotic cell lines
☒	☐ Palaeontology and archaeology
☒	☐ Animals and other organisms
☒	☐ Clinical data
☒	☐ Dual use research of concern
☒	☐ Plants

Methods

n/a	Involved in the study
☒	☐ ChIP-seq
☒	☐ Flow cytometry
☒	☐ MRI-based neuroimaging
